# The effects of stress on avoidance in rodents: An unresolved matter

**DOI:** 10.3389/fnbeh.2022.983026

**Published:** 2022-09-28

**Authors:** Alba López-Moraga, Tom Beckers, Laura Luyten

**Affiliations:** ^1^Center for the Psychology of Learning and Experimental Psychopathology, Faculty of Psychology and Educational Sciences, KU Leuven, Leuven, Belgium; ^2^Leuven Brain Institute, KU Leuven, Leuven, Belgium

**Keywords:** active avoidance, passive avoidance, inhibitory avoidance, chronic stress, acute stress, stress induction, rodents, anxiety-related disorders

## Abstract

In the face of a possible threat, a range of physiological (e.g., increased heart rate) and behavioral (e.g., avoidance or escape) responses are recruited. Here, we will focus on avoidance, in its persistent form one of the core symptoms of anxiety disorders and obsessive-compulsive disorder. The initial goal of fear and avoidance responses is to increase survival, but if they become persistent or overgeneralize, they can disrupt normal daily functioning, and ultimately even result in anxiety-related disorders. Relatedly, acute stress responses promote adaptation and survival, while chronic stress has been found to aggravate pathophysiology. Thus, stress might trigger the transition from adaptive to maladaptive responses, e.g., from goal-directed to persistent avoidance. Animal models are prime tools to unravel if and how stress influences avoidance. This is typically done by performing stress inductions prior to the assessment of (passive or active) avoidance behavior. Despite its clinical relevance, the current literature on this topic is fragmented, and an overall conclusion is lacking. In this Review, we first recapitulate the state of the art regarding stress and active as well as passive avoidance procedures. We then summarize the behavioral effects of acute and chronic stress on active and passive avoidance, and discuss the main neurobiological findings of the field. Finally, we highlight possible reasons for the largely contradictory findings in the literature and we propose strategies to further unravel the effect of stress on avoidance behavior. A deeper understanding of this currently unresolved matter may provide further insights in the etiology and treatment of anxiety-related disorders.

## Introduction

Anxiety-related disorders are the most prevalent mental health conditions (Craske and Stein, 2016). They are typically characterized by an excessive fear and persistent avoidance of perceived threats. Persistent avoidance, in particular, while an important coping mechanism for individuals with an anxiety disorder, also represents one of its most debilitating symptoms. Avoidance can be defined as a response that increases the distance between an individual and a (perceived or actual) threat or aversive event. The avoidance response can be either external (e.g., a person suffering from acrophobia who avoids heights) or internal (e.g., a person suffering from post-traumatic stress disorder who attempts to suppress thoughts or feelings related to the traumatic event). Avoidance is not a detrimental behavior *per se*. When an individual faces an acute threat and performs a goal-directed action (e.g., choosing longer but safer roads in difficult weather conditions), this promotes survival. However, avoidance can become maladaptive when an individual shuns situations that are objectively safe, but that are perceived as threats by that individual (Arnaudova et al., 2017). Currently, the triggers that drive a transition from adaptive to maladaptive avoidance are not fully understood, but one of the candidates appears to be stress.

A novel, unexpected or threatening stimulus (i.e., a stressor) will activate the hypothalamic-pituitary-adrenal (HPA) axis and trigger the release of glucocorticoids (GCs, cortisol in humans and corticosterone in rodents) (McEwen and Akil, 2020). As is the case for avoidance, an acute stress response can promote survival, but its chronification can lead to disease through neural, cardiovascular, immune, autonomic, and metabolic effects (McEwen, 2008). First, GCs are involved in short-term adaptive responses to threats, including the mobilization of glucose into the bloodstream, an increase in cardiovascular tone and the interruption of functions that are not essential for short-term survival (e.g., feeding, reproduction). In addition, GCs can induce more long-term responses, including effects on learning and memory, such as enhanced consolidation of new memories and reduced expression of extinction (De Quervain et al., 2016). A healthy response to an acute stressor requires rapid GC synthesis, but also an effective termination of the stress response to limit the actions of these hormones. However, if stress becomes chronic, the same mediators that were implicated in promoting short-term adaptations to increase survival can eventually cause permanent damage (McEwen and Akil, 2020).

Given the important roles that stress and avoidance have in psychopathology, investigating their relationship in the lab can provide clinically valuable insights. Non-human animal models (from this point on referred to as animal models) can help us to achieve this, especially given how well-conserved the stress response is between rodents and humans. Eventually, more insight into the potential dysregulation of avoidance by stress may help us to better understand the pathophysiology of anxiety-related disorders and to devise novel interventions and molecular targets (Bale et al., 2019).

Early experiments with dogs (Overmier and Seligman, 1967; Seligman and Maier, 1967) paved the way for dozens of rodent studies investigating the relationship between avoidance and stress. After several decades of research on the topic, it is clear that there is a complex relationship between stress and avoidance that can be modulated by a variety of factors, such as the nature of the stressor and an individual’s sex, amongst others. In this review, we will delve into those factors and consolidate a literature that is at present rather fragmented. We will start with an overview of the dominant stress induction and avoidance procedures that have been used in this field of research. Next, we will discuss the main behavioral observations as well as the neurobiological mechanisms that have been put forward.

## Methodology

### Literature search

We performed an initial literature search in the PubMed database using the search term [“Stress, Psychological” (Mesh) AND Avoidance] in November 2021, which generated 4,675 results. An email alert using this search query was then generated and fitting published studies were considered for the review until March 2022. Moreover, additional studies were inspected based on the reference lists of the reviewed studies. Studies were considered for inclusion in the review if they investigated the effect of a stress induction procedure on an avoidance task in rodents, and contained an appropriate control condition. Finally, a total of 38 studies was included.

### Stress induction procedures

**FIGURE 1:**
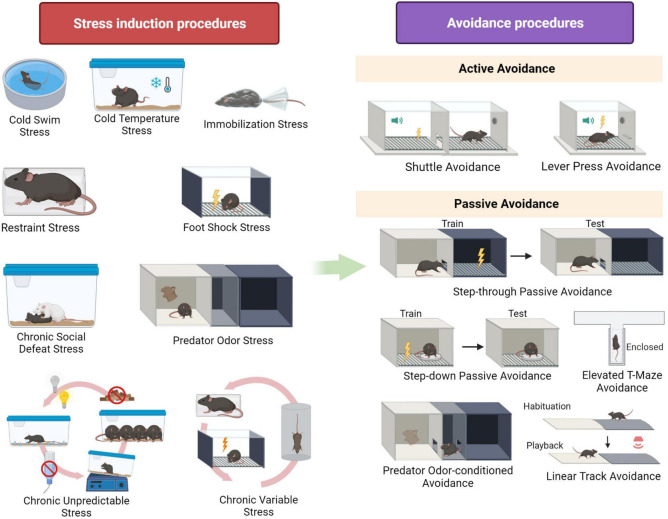
Overview of stress induction procedures and avoidance procedures mentioned in the review. Created with BioRender.

To examine the effects of stress on avoidance in the laboratory, first a (chronic or acute) stress situation is presented, followed by an avoidance task. We refer to a stress procedure as being acute if the stressor is applied just once, while chronic stress refers to a repeated application of a stressor. Various procedures to induce acute and chronic stress exist across the rodent literature, and an extensive review of all different behavioral and non-behavioral models can be found elsewhere (e.g., Atrooz et al., 2021). Here, we will focus specifically on the procedures that have been used to study the effects of stress on avoidance. Figure 1 presents a visual overview of these stress induction procedures (left panel).

#### Cold swim stress

The rodent is placed in cold water (at 4°C) and left to swim for a few minutes (Weiss and Glazer, 1975; Weiss et al., 1975).

#### Cold temperature stress

Instead of using cold water to induce cold stress, here the temperature of the rodent’s environment is reduced. The rodent is exposed to alternating 1-h intervals of normal temperature (24°C) and cold temperature (−3°C) from 9 am to 4 pm and cold temperature from 4 pm to 9 am for 5 days (Hata et al., 1989).

#### Cold bath and immobilization stress

The rodent is immobilized by fixating its front and hind limbs with adhesive plaster and restrained using a vertical plastic mesh. It is then immersed in a cold water bath (22°C) for an hour (Klenerová et al., 2003a,b).

#### Immobilization stress

The rodent is immobilized for 2 h per day during 10 days using a disposable plastic rodent restrainer (Wood et al., 2008).

#### Restraint stress

The rodent is placed in a small adjustable Plexiglass cylinder with small holes to allow for ventilation. The duration of this stress induction procedure can range from a single acute exposure of 30 min to chronic restraint sessions of 10 h for several weeks (Gamaro et al., 1999; Yamada et al., 2003; Nooshinfar et al., 2011; de Andrade et al., 2012; Radahmadi et al., 2013; Navarro-Francés and Arenas, 2014; Kozlova et al., 2021; Shukla and Chattarji, 2021; Wang et al., 2022).

#### Shock stress

The rodent is placed in a chamber with an electrified grid floor that can deliver foot shocks or researchers place a tail electrode on the rodent’s tail to deliver tail shocks. The duration of the shock stress sessions (from 5 min to 2 h), number of shocks (from 60 to 80 per session) and the intensity of the shocks (from 0.6 to 2 mA) vary between experiments. For chronic stress induction, the number of sessions ranges from 4 days to several weeks (Seligman et al., 1975; Weiss and Glazer, 1975; Weiss et al., 1975; Jodar et al., 1996; Lehmann et al., 1999; Koba et al., 2001; Brennan et al., 2005; Wakizono et al., 2007).

#### Chronic social defeat stress

The experimental rodent, denoted as intruder, is placed in the home cage of a resident, which is usually a rodent from a bigger and more aggressive strain or an older rodent selected based on their aggressive behavior. Resident-intruder encounters range from 5 to 10 min and take place daily for 3 weeks (Monleón et al., 2015; Duque et al., 2016, 2017).

#### Unpredictable chronic mild stress

The rodent is presented with different mild stressors daily for 2 weeks. Examples of these stressors are food or water deprivation overnight, damp bedding, isolation, or short restraint stress sessions (de Andrade et al., 2013).

#### Chronic variable stress

In this modification of UCMS, the stressors have a higher intensity, and the number of sessions ranges from 12 to 40 days. Examples of stressors are forced cold stress swimming, and food and water deprivation for 18 to 24 h (Wakizono et al., 2007; Dos Santos et al., 2017).

### Avoidance procedures

To examine avoidance behavior in rodents, different procedures have been used, which can generally be divided into two classes: active and passive avoidance tasks.

### Active avoidance

In active avoidance procedures, an antecedent stimulus is presented first and followed by an aversive event unless the animal performs a specific response (e.g., a tone is presented and if the rodent goes to the opposite compartment of the test chamber before the tone ends, foot shock is withheld) (Krypotos et al., 2015). Figure 1 (right panel) provides an overview of the different rodent active avoidance protocols that will be described in this review.

#### Shuttle avoidance

The rodent is placed in a box with an electrified grid floor and two compartments that are separated with a door. First, a training session takes place, which usually starts with a habituation period of a few minutes. Afterward, a tone or a light (or both) is presented and followed by a foot shock, which continues until the rodent crosses to the opposite compartment (intensity of foot shocks ranges from 0.5 to 1.25 mA, often with a maximum duration of 15 s). The number of trials presented per session varies between 25 and 80, depending on the experiment (Glazer et al., 1975; Weiss and Glazer, 1975; Weiss et al., 1975; Gamaro et al., 1999; Lehmann et al., 1999; Koba et al., 2001; Wakizono et al., 2007).

#### Lever press and nose poke avoidance

A rodent is placed in a box with an electric grid floor or fitted with a tail electrode that can deliver a shock. After habituation, a tone or a light is presented for 5 s and, unless a nose poke or a lever press is performed during these 5 s, an electric shock of 1 mA is delivered. The shock can still be terminated by a nose poke or lever press during shock delivery, but this is considered an escape rather than an avoidance response (Weiss and Glazer, 1975; Brennan et al., 2005).

### Passive avoidance

In contrast with active avoidance, in passive avoidance procedures (also referred to as inhibitory avoidance) an aversive event occurs unless an animal refrains from a certain response (e.g., if a rodent moves from a light to a dark chamber, it receives a foot shock) (Krypotos et al., 2015). Figure 1 (right panel) presents an overview of the passive avoidance procedures described in this review.

#### Step-through passive avoidance

This task takes place in a shuttle box with a light compartment and a dark one which has an electrified grid floor. Both compartments are separated by a wall that has a small door allowing rodents to shuttle between compartments. In the training phase, the rodent is placed in the light compartment facing away from the dark one. When the rodent crosses to the dark compartment, it receives a single foot shock varying between 3 and 5 s and between 0.5 mA and 4 mA, depending on the study. The test trial (without shock delivery) takes place 24 h later. The rodent is again placed in the light compartment and the latency to cross to the dark compartment is recorded (Jodar et al., 1996; Klenerová et al., 2003b,a; Yamada et al., 2003; Nooshinfar et al., 2011; Radahmadi et al., 2013; Navarro-Francés and Arenas, 2014; Monleón et al., 2015; Duque et al., 2016, 2017; Kozlova et al., 2021; Wang et al., 2022).

#### Step-down passive avoidance

During training, the rodent is placed on an elevated platform from which the rodent can step down onto an electrified grid that delivers a foot shock (ranging from 0.5 mA to 5 mA). One day later, during the test session (without shock delivery), the rodent is again placed on the elevated platform and the latency to step down is measured (Nishimura et al., 1989; Dos Santos et al., 2017).

#### Elevated T-maze avoidance

During training, the rodent is exposed to one of the open arms of an elevated T-maze (ETM) for 30 min. On the next day, the test session begins with the rodent being placed at the end of the enclosed arm and measuring its latency to reach the open arm. Two to three avoidance test sessions are usually performed. A variant of the task allows to test for escape behavior by placing the animal in an open arm during test (Wood et al., 2008; de Andrade et al., 2012, 2013).

#### Predator odor-conditioned avoidance

On day 1, the rodent is allowed to inspect three chambers with different tactile and visual cues. The chamber where the rodent spends most of the time is excluded for the next sessions. On day 2, the rodent again explores the two non-excluded chambers for 5 min. On day 3, the rat is placed in one chamber without odor (counterbalanced across animals) for 15 min, and on day 4, it is placed in the opposite chamber for 15 min, which now has the odor of bobcat urine (a soaked sponge is placed under the floor of the chamber). On day 5, the rat is allowed to explore the two compartments (one previously paired with predator odor and one that was not) for 5 min and the time spent in each compartment is recorded (Whitaker and Gilpin, 2015; Whitaker et al., 2016; Schreiber et al., 2017; Albrechet-Souza et al., 2020; Weera et al., 2020, 2021).

#### Linear track avoidance

The rodent is placed at the center of a linear track and a 5-min habituation phase takes place. Subsequently, a 3-min auditory stimulus (e.g., 22–Hz aversive ultrasonic vocalization) is presented twice, at one side of the track, with a 5-min silent interval. After the second playback, the rodent is allowed to explore the track for another 5 min before returning to its home cage. Time spent on the distal half compared to proximal half of the track (relative to the speaker) is compared during habituation and playback (Shukla and Chattarji, 2021).

## Behavioral studies

This section provides an overview of the documented behavioral effects of stress on avoidance, as examined with the above-mentioned procedures (see Table 1 for a summary of these behavioral results). The results are organized according to the duration of the stressor (acute or chronic) and the type of avoidance (active or passive).

**TABLE 1:** Overview of the effect of stress on avoidance.

Acute stress and active avoidance					
Stressor	Avoidance task	Animal	Sex	Effect of stress on avoidance	Reference
Shock	Lever press	Rats (Sprague-Dawley)	Male	↓	(Seligman et al., 1975)
Shock	Shuttle box	Rats (Sprague-Dawley)	Male	↓	(Weiss and Glazer, 1975)
Cold swim	Shuttle box	Rats (Sprague-Dawley)	Male	↓	(Weiss and Glazer, 1975)
Shock	Shuttle box	Rats (Wistar)	Male	↓	(Lehmann et al., 1999)
Cold swim	Nose poke	Rats (Sprague-Dawley)	Male	=	(Weiss and Glazer, 1975)
Shock	Nose poke	Rats (Sprague-Dawley)	Male	=	(Weiss and Glazer, 1975)
Shock	Lever press	Rats (Wistar)	Male	= (1 s shock in active avoidance)	(Brennan et al., 2005)
Shock	Lever press	Rats (Wistar)	Male	↑ (30 s shock in active avoidance)	(Brennan et al., 2005)
Shock	Shuttle box	Rats (Wistar)	Male	↑	(Koba et al., 2001)
Shock	Shuttle box	Rats (Wistar, Fisher 344, Lewis)	Male	↑	(Wakizono et al., 2007)

**Chronic stress and active avoidance**					

**Stressor**	**Avoidance task**	**Animal**	**Sex**	**Effect of stress on avoidance**	**Reference**

Restraint	Shuttle box	Rats (Wistar)	Female	↓	(Gamaro et al., 1999)
Cold swim	Shuttle box	Rats (Sprague-Dawley)	Male	=	(Weiss et al., 1975)
Shock	Shuttle box	Rats (Sprague-Dawley)	Male	=	(Weiss et al., 1975)
Variable stress	Shuttle box	Rats (Wistar, Fisher 344, Lewis)	Male	=	(Wakizono et al., 2007)
Cold temperature	Shuttle box	Rats (Wistar)	Male	↑	(Hata et al., 1989)

**Acute stress and passive avoidance**					

**Stressor**	**Avoidance task**	**Animal**	**Sex**	**Effect of stress on avoidance**	**Reference**

Restraint	Step-through	Mice (CD1)	Female	↓ (if high anxiety)	(Navarro-Francés and Arenas, 2014)
Restraint	Step-through	Rats (Wistar)	Male	↓ (3 h or 5 h stress)	(Nooshinfar et al., 2011)
Cold swim + restraint	Step-through	Rats (Wistar)	Male	↓ (stressor 1 h before avoidance)	(Klenerová et al., 2003a)
Cold swim + restraint	Step-through	Rats (Wistar)	Male	= (stressor 4 h before avoidance)	(Klenerová et al., 2003a)
Restraint	Step-through	Rats (Wistar)	Male	= (1 h stress)	(Nooshinfar et al., 2011)
Restraint	Step-through	Mice (C57BL/6J)	Female	=	(Yamada et al., 2003)
Restraint	Elevated T-maze	Rats (Wistar)	Male	↑	(de Andrade et al., 2012)
Restraint	Step-through	Mice (CD1)	Male	↑ (if high anxiety)	(Navarro-Francés and Arenas, 2014)
Shock	Step-through	Mice (ddY)	Male	↑	(Jodar et al., 1996)

**Chronic stress and passive avoidance**					
**Stressor**	**Avoidance task**	**Animal**	**Sex**	**Effect of stress on avoidance**	**Reference**

Cold temperature	Step-down	Mice (ddY)	Male	↓	(Nishimura et al., 1989)
Restraint	Linear track	Rats (Sprague-Dawley)	Male	↓	(Shukla and Chattarji, 2021)
Restraint	Step-through	Mice (C57BL/6J)	Male	↓	(Wang et al., 2022)
Restraint	Step-through	Rats (Wistar)	Male	↓	(Radahmadi et al., 2013)
Variable stress	Step-down	Rats (Wistar)	Male	↓	(Dos Santos et al., 2017)
Social defeat	Step-through	Mice (CD1)	Male	↓ (10 min stress)	(Monleón et al., 2015)
Social defeat	Step-through	Mice (CD1)	Male	↓ (if susceptible)	(Duque et al., 2016, 2017)
Restraint	Step-down	Rats (Wistar)	Female	=	(Gamaro et al., 1999)
Restraint	Step-through	Mice (C57BL/6J)	Male	=	(Kozlova et al., 2021)
Shock	Step-through	Mice (ddY)	Male	=	(Jodar et al., 1996)
Immobilization	Elevated T-maze	Rats (Sprague-Dawley)	Male	↑	(Wood et al., 2008)
Restraint	Step-through	Rats (Wistar)	Male	↑	(Nooshinfar et al., 2011)
Unpredictable Mild Stress	Elevated T-maze	Rats (Wistar)	Male	↑	(de Andrade et al., 2013)
Social defeat	Step-through	Mice (CD1)	Male	↑ (5 min stress)	(Monleón et al., 2015)

↓: stressed animals show less avoidance than controls; = : stressed animals show no significant difference with controls; ↑: stressed animals show higher avoidance than controls.

### Acute stress and active avoidance

As mentioned before, Seligman and Maier (1967) were among the first to investigate how stress affects avoidance responding. They replicated their initial results with dogs in rats, showing that acute shock stress impaired performance in the active avoidance task. Stressed rats showed reduced lever pressing to avoid a shock compared to controls (Seligman et al., 1975). Other researchers replicated these results using shock stress or cold swim stress preceding a shuttle active avoidance task (Weiss and Glazer, 1975; Lehmann et al., 1999).

Weiss and Glazer (1975) performed further experiments in which they replaced the shuttle active avoidance task with a nose poking task, and, in contrast with their prior findings, observed no differences between controls and stressed animals, regardless of the type of stressor (cold or shock stress). Similar results were obtained using a lever press active avoidance task after rats went through tail shock acute stress. There, stressed animals, and non-stressed controls performed similar avoidance responses in a shuttle task with 1-s shocks (Brennan et al., 2005).

In contrast with the above-mentioned studies that found either reduced avoidance or no significant effects of stress, a more recent study found that acutely shock-stressed animals showed increased rather than reduced avoidance responding (Koba et al., 2001). Comparable results were obtained using a lever press active avoidance task after rats went through acute tail shock stress. Stressed animals performed more avoidance responses than controls when shocks in the shuttle task lasted for 30 s (Brennan et al., 2005). Wakizono et al. (2007) compared the effects of acute shock on a subsequent test of active avoidance in different strains (with different stress vulnerability due to distinct genetic background) and found that acute inescapable shock significantly increased avoidance responses in all three strains (Wakizono et al., 2007).

In summary, the effects of acute stress on active avoidance are inconsistent. Contradictory effects (i.e., increased, reduced or unchanged avoidance) have been found, even with highly similar procedures.

### Chronic stress and active avoidance

Gamaro et al. (1999) found that, after chronic restraint, stressed animals showed less avoidance responses than a non-stressed control group. Several other studies, however, failed to find an effect of chronic stress (be it cold swim, shock, or variable stress) on avoidance in the shuttle box (Weiss et al., 1975; Wakizono et al., 2007). In contrast with these findings, others found that rats stressed with cold temperature showed higher avoidance than controls, with stressed rats shuttling more often than controls during the presentation of the warning signal (Hata et al., 1989).

In conclusion, like for acute stress, the results of chronic stress on active avoidance in the shuttle box are variable. Moreover, the limited studies that are available are difficult to compare, as they all used different types of stressors.

### Acute stress and passive avoidance

Researchers have tried to address the question of how acute stress affects passive avoidance responses using different strategies. Navarro-Francés and Arenas (2014) investigated if there are individual differences in the relationship between acute restraint stress and passive avoidance. First, mice were classified as high, mid, or low-anxious animals depending on the time they spent in the open arms of an elevated plus maze. Next, half of the animals were restrained, after which they all performed the avoidance task. Acute stress had effects on avoidance behavior in the high-anxious animals only and, moreover, these effects were sex-dependent. High-anxious males showed increased inhibitory avoidance (i.e., a higher latency to enter the dark compartment compared to non-stressed controls), while high-anxious females showed reduced inhibitory avoidance after stress induction. Other researchers have investigated the effects of longer compared to shorter restraint stress sessions in the step-through avoidance procedure. Nooshinfar et al. (2011) found that restraint sessions of 3 or 5 h impaired subsequent avoidance, such that stressed rats showed a shorter latency to enter the compartment where they were previously shocked. On the contrary, a shorter restraint session of 1 h had no effect on avoidance acquisition. Another approach using combined stressors (restraint stress and cold bath) supports the notion that prior stress can produce an impairment in avoidance (Klenerová et al., 2003a). Rats stressed 1 h before avoidance training or stressed after training showed shorter latencies than controls for stepping into the dark compartment where they were previously shocked. However, if the stress was terminated 4 h before avoidance training, no differences with control animals were observed, and likewise, if stress was postponed until 3 h after training, it did no longer have an effect on avoidance in the test session (Klenerová et al., 2003a). Then again, in a study with female mice, no significant differences were found between restrained and control mice in step-through passive avoidance (Yamada et al., 2003).

Although the studies above mostly found either no effect or reduced avoidance in stressed rodents, there have been reports of acute stress increasing passive avoidance in rodents. In a study with acute restraint, stressed rats had higher latencies to explore the open arms of a T-maze compared to controls (de Andrade et al., 2012). Another study exposed mice to acute shock stress, and found that stressed animals showed increased latencies in crossing to the dark compartment in the step-through avoidance task (Jodar et al., 1996).

After reviewing these studies, we can conclude that the effects of acute stressors on passive avoidance may depend on when the stressor is applied in regards to the passive avoidance test, on the duration of the applied stressor or even pre-experimental differences in anxiety and sex of the animals. Currently, it is difficult to draw an overarching conclusion regarding the effects of acute stress on passive avoidance.

### Chronic stress and passive avoidance

Several studies have reported that chronic stress reduces the learning and expression of passive avoidance in rodents. Nishimura et al. (1989) reported that mice going through repeated cold temperature stress had an impairment in acquiring avoidance, with low latencies during test to cross to a dark compartment where they were previously shocked. Shukla and Chattarji (2021) used a novel procedure to test passive avoidance in a linear track and they reported that restrained rats showed less avoidance than control animals. Similarly, researchers have found that extensive chronic stress impairs step-through passive avoidance in mice (Wang et al., 2022). In a study investigating the effects of exercise combined with chronic restraint stress, it was found that exercise alone had beneficial effects on the acquisition of avoidance. However, stressed rats (with or without the exercise intervention) showed memory impairments in the avoidance task. Thus, stressed rats (regardless of the exercise intervention) had a lower latency to enter a compartment where they were previously shocked (Radahmadi et al., 2013). Other work reported that rats that were allowed to exercise during their adolescence and were subjected to chronic variable stress (CVS) during adulthood, performed better in the passive avoidance task than rats that did not have a history of exercise (Dos Santos et al., 2017). Mice that went through high chronic social defeat stress (CSDS) showed a lower latency in the passive avoidance task (Monleón et al., 2015; Duque et al., 2016). Additionally, when further investigating differences between CSDS-resilient compared to susceptible mice, it was found that passive avoidance was lower in the susceptible than in the resilient group (Duque et al., 2017).

In contrast with all of these studies showing that chronic stress impairs passive avoidance responding, others did not find significant differences in avoidance between stressed and control animals. For instance, Kozlova et al. (2021) found no differences between restrained and control mice, and Gamaro et al. (1999) found the same in female rats. Likewise, Jodar et al. (1996) found no significant differences in passive avoidance between stressed and control animals when using shock as a chronic stressor.

It is important to note that there are also studies that reported results in the opposite direction, with chronic stress facilitating passive avoidance responses. One study indicated that immobilization stress increased avoidance in the ETM, as stressed rats had higher latencies to move from the enclosed arm of the T-maze to the open arms (Wood et al., 2008). Nooshinfar et al. (2011) found that after chronic restraint stress (2 h per day during 1 week), stressed rats showed increased latencies in the avoidance test, meaning that avoidance was elevated relative to controls. Using uncontrollable chronic mild stress (UCMS), others found that stress heightened avoidance performance in the ETM, as stressed rats showed a lower latency to move from the enclosed arm of the T-maze to the open arm compared to controls (de Andrade et al., 2013). In contrast with mice that went through high CSDS (see above), mice that went through moderate CSDS (5-min sessions) showed longer step-through latency compared to controls, suggesting that their memory was not affected negatively.

So, whereas about half of the reported studies support that chronic stress reduces passive avoidance responses, especially studies using the step-through task, the effects may depend on the duration of the stress sessions. Also, studies using an alternative passive avoidance task, such as the ETM, which measures the expression of innate, instinctive avoidance behavior rather than the acquisition and expression of a newly trained avoidance response, have typically reported increased, rather than impaired, avoidance after chronic stress.

In conclusion, the behavioral effects of acute and chronic stress on avoidance are not straightforward. Different types of stressors, strains and sex may affect avoidance very differently and there are many contradictory results. Researchers need to plan carefully which type of stressor and also for which duration (acute or chronic stress, but also length of sessions), as well as at which timepoint of life they want to apply a stressor or an intervention. Moreover, contradictory results found in male and female animals highlight the importance to include animals of both sexes, since results may not be translatable across sexes.

## Neurobiological mechanisms

**FIGURE 2:**
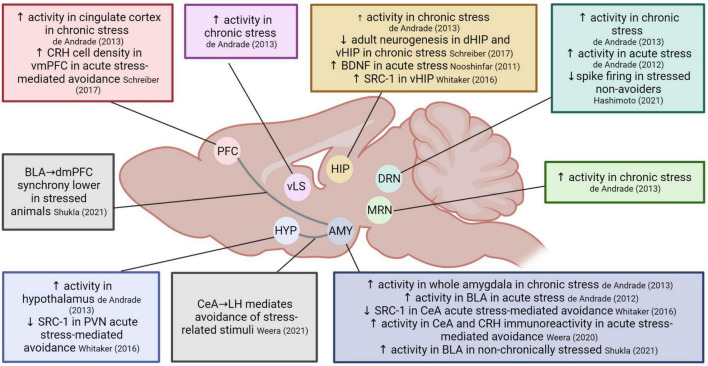
Overview of the neurobiological mechanisms involved in the effects of stress on avoidance. Several neurotransmitter systems have been implicated, including noradrenergic (Glazer et al., 1975; Driscoll et al., 1983; Nishimura et al., 1989), serotonergic (Driscoll et al., 1983; Yamada et al., 2003; Hashimoto et al., 2021), dopaminergic, cholinergic and GABAergic transmission (Nishimura et al., 1989). ACTH, adrenocorticotropic hormone; AMY, amygdala; BDNF, brain-derived neurotrophic factor; BLA, basolateral amygdala; CeA, central nucleus of the amygdala; CORT, corticosterone; CRH, corticotropin-releasing hormone; dHIP, dorsal hippocampus; dmPFC, dorsomedial prefrontal cortex; DRN, dorsal raphe nuclei; HIP, hippocampus; HYP, hypothalamus; LH, lateral hypothalamus; MRN, median raphe nucleus; PFC, prefrontal cortex; PVN, paraventricular nucleus; SRC-1, steroid receptor co-activator-1; vHIP, ventral hippocampus; vLS, ventrolateral septum; vmPFC, ventromedial prefrontal cortex. Created with BioRender.

In this section, we present the main molecular and circuit findings regarding the effects of stress on avoidance behavior (see Figure 2 for a summary).

Glazer et al. (1975) performed one of the first molecular studies on the topic and found that deficits in avoidance and escape behavior were mediated by a reduction in noradrenergic activity, which has been confirmed in later research. For example, Driscoll et al. (1983) tested Roman high (RHA) versus low avoidance (RLA) rats in a shuttle avoidance procedure involving 3 different conditions: control, inescapable shock, and avoidance, and measured the levels of different molecules in selected brain areas. Rats exposed to inescapable shocks showed reduced noradrenaline levels. In addition, there were differences in serotonin levels after inescapable shock: RLA rats showed an increased serotonin metabolism in the hypothalamus, whereas RHA rats showed the opposite. Additionally, RHA rats in the avoidance condition showed a reduction in serotonin in the cortex, hippocampus, and hypothalamus. Together, these results suggest an important role for serotonin in stress and avoidance behavior (Driscoll et al., 1983). Later work confirmed the potential importance of the serotonergic system by using mice lacking neuromedin B, which is a peptide involved in the stress response. Neuromedin B-deficient mice showed altered anxiety-related behavior (e.g., in the elevated-plus maze) and abnormal serotonin functioning. Moreover, deficient female mice that were previously exposed to restraint stress showed reduced passive avoidance, with low latencies to step through a compartment where they were previously shocked (Yamada et al., 2003).

Not only serotonin but also dopaminergic, noradrenergic, cholinergic, and GABAergic signaling have been implicated in the relation between stress and avoidance. Nishimura et al. (1989) reported that mice going through chronic cold temperature stress had an impairment in acquiring passive avoidance, with low latencies during test to cross to a dark compartment where they were previously shocked. Notably, this deficit was reverted with a single injection of dopamine and noradrenaline blockers (chlorpromazine and carpipramine) or by repeated administration of drugs that activate the GABAergic system (Neurotropin^®^) or the GABAergic and cholinergic systems (calcium hopantenate). In contrast, the deficit in passive avoidance could not be reversed through single or repeated administration of benzodiazepines (alprazolam and diazepam) (Nishimura et al., 1989). A study using repeated foot shocks as a stress induction (Jodar et al., 1996) rather than cold temperature stress, observed that scopolamine (an anticholinergic drug) administered 30 min before training caused impairment of passive avoidance during test, but had no effect in animals that received the stress induction before or after training (Jodar et al., 1996).

A range of studies have examined the effects of a variety of other drugs on avoidance in stressed rodents. This includes studies describing the attenuating effects of antidepressants and anti-inflammatory drugs on stress-heightened passive avoidance (Duque et al., 2016, 2017). Other authors report rescuing impaired avoidance *via* HPA axis inhibition (with tenuifolin) (Wang et al., 2022). Yet others reported differences in benzodiazepine receptors in the brains of stressed animals (Lehmann et al., 1999) and increases in hippocampal BDNF in stress-impaired passive avoidance (Nooshinfar et al., 2011). However, all of the results mentioned in this paragraph have not yet been followed up or replicated in the literature.

Unsurprisingly, given the role of corticosteroids (CORT) in stress reactivity, a number of studies have documented a role for GC-related signaling in stress and avoidance. In a recent study, Varejkova et al. (2018) used a three-chamber apparatus to test the relationship between stress and avoidance: one chamber was the neutral zone, a middle chamber was the entrance zone and a last chamber was the aversive zone, which contained a restraining tube. Using this task, they reported that non-stressed mice lacking the corticotropin-releasing hormone (CRH) gene showed more avoidance than non-stressed wild-types, i.e., a shorter latency to enter the neutral area and spending significantly more time in the neutral chamber (Varejkova et al., 2018). Interestingly, acutely restraint-stressed CRH-deficient and acutely restraint-stressed wild-type animals showed similar avoidance of the aversive area of the arena regardless of CRH-deficiency, spending both significantly less time in the aversive area than non-stressed controls (Varejkova et al., 2018). A different study showed that a deficit in passive avoidance induced by stresscopin (a selective ligand for CRHR2) mimicked the deficit induced by a combination of restraint and cold bath stress. In addition, combining stresscopin and behavioral stressors produced an even stronger impairment of the acquisition of passive avoidance (Klenerová et al., 2003b). The role of stress hormones has also been investigated using the predator odor conditioned-avoidance paradigm. Note that these studies are somewhat different from other work described in this review in that they do not involve a separate stress induction procedure prior to avoidance learning and/or testing. Using the predator odor conditioned-avoidance procedure, researchers have reported an inverse relationship between adrenocorticotropic hormone (ACTH) and CORT concentrations and avoidance of the predator-odor-paired chamber. Specifically, in comparison with subjects with a normal HPA response, animals that exhibited a blunted ACTH and CORT response to the predator odor exposure were more likely to be classified as avoiders (rats that showed a > 10 s decrease in time spent in the odor-paired chamber were classified as “avoiders,” while all the rest were considered “non-avoiders”) (Whitaker and Gilpin, 2015). A follow-up experiment found no difference in magnitude of avoidance between males and females, but females did exhibit a lower startle response and an immediate increase in CORT levels after predator odor exposure compared to males (Albrechet-Souza et al., 2020). Additionally, it has been reported that CORT pretreatment reduces the magnitude and incidence of avoidance in the predator-odor paired context, but this was tested in males only (Whitaker et al., 2016).

In summary, several studies support the implication of noradrenaline (Glazer et al., 1975; Driscoll et al., 1983) and serotonin (Driscoll et al., 1983) in the alteration of avoidance responses in stressed animals. Other pharmacological studies implicated dopamine, GABA, or cholinergic systems (Nishimura et al., 1989). It should be noted that some of these studies used drugs that have widespread effects on several neurotransmitter systems. Future studies using more precise techniques can help us to dissect which neuronal populations and brain regions exactly mediate the effects of acute or chronic stress on avoidance behavior.

Besides the pharmacological studies mentioned above, more recent research has focused on identifying the brain regions involved in enhancing or impairing avoidance after stress induction.

One study reports that acutely restrained animals undergoing the ETM avoidance task (and exhibiting elevated avoidance) showed increased levels of Fos, a marker of neural activity, in the basolateral amygdala and the dorsal raphe nucleus (DRN) (de Andrade et al., 2012). Chronically stressed animals in the ETM task (also exhibiting elevated avoidance) showed the same increased activity in the DRN, but also increased activity in the whole amygdala, as well as in the median raphe nucleus, the hippocampus, hypothalamus, ventrolateral septum, and cingulate cortex. Additionally, these animals showed a decrease in dorsal and ventral hippocampal adult neurogenesis compared to controls. Overall, this pattern of activity did not overlap with the increased Fos expression seen during stress (in the cingulate cortex, periaqueductal gray, and locus coeruleus) (de Andrade et al., 2013). Together, these results suggest that acute and chronic stress alter avoidance behavior in the ETM task similarly while they engage common (basolateral amygdala and DRN) but also different areas in the brain related to the HPA axis.

In line with the abovementioned observations of DRN involvement in stress and avoidance behavior, a recent study compared electrophysiological properties of serotonin neurons in the ventral DRN in stressed and non-stressed rats. Animals were exposed to inescapable shock and 24 h later went through avoidance training, where they could stop a foot shock by pressing a lever. Animals that failed to learn this avoidance behavior showed attenuated spike firing compared to naive rats and stressed animals that did learn to avoid (Hashimoto et al., 2021).

Studies using the predator odor stress paradigm followed by a place preference test in the same context support the ETM findings described above regarding the involvement of the amygdala, but also zoomed in on concrete circuitry and mechanisms that may be involved in mediating avoidance in stressed animals. Stress-heightened avoidance was associated with lower SRC-1 (steroid receptor co-activator-1, involved in the regulation of GR-mediated CRH gene transcription) expression in the PVN and the central amygdala (CeA), but higher expression in the ventral hippocampus. Moreover, changes in SRC-1 expression correlated with avoidance behavior (Whitaker et al., 2016). A later study confirmed the involvement of the CeA and found higher numbers of cFos + cells and higher CRH immunoreactivity in the CeA in avoiders compared to non-avoiders (Weera et al., 2020). Additionally, it has been reported that the CeA projection to the lateral hypothalamus is preferentially active in avoiders compared to non-avoiders, and that its chemogenetic inhibition reduces avoidance in the predator odor-paired context. Finally, chemogenetic activation of the same projection produced conditioned place avoidance in naïve rats, further supporting the importance of this circuit for mediating persistent avoidance following acute stress exposure (Weera et al., 2021). In addition to the findings in CeA, avoiders also showed higher CRH cell density in the ventro-medial prefrontal cortex (vmPFC), which was positively correlated with the magnitude of avoidance. Furthermore, infusion of CRH in vmPFC produced conditioned avoidance of the paired context, whereas infusion of a CRHR1 antagonist in vmPFC reduced avoidance behavior (Schreiber et al., 2017).

A recent study reported that prefrontal cortex (PFC) projections to BLA mediate active avoidance (Diehl et al., 2020). In line with this finding, Shukla and Chattarji (2021) investigated the involvement of BLA-dorsomedial PFC (dmPFC) communication in avoidance of stressed animals. Chronically restrained and control rats were exposed to aversive ultrasonic vocalizations (USVs) on a linear track. Whereas controls avoided the area closest to the speaker that emitted the aversive calls, stressed animals did not. They found that non-stressed control rats had higher activity in the BLA than stressed rats and that inactivation of the BLA in non-stressed controls impaired avoidance. Finally, *in vivo* recordings showed that theta waves in BLA and in dmPFC were increased in controls during exposure to the aversive USV calls, but not in stressed rats. The authors stated that the increased BLA-dmPFC synchrony seen in control rats may reflect communication between both areas thereby mediating avoidance responses, which may be impaired in stressed animals (Shukla and Chattarji, 2021).

We conclude that, despite the variability in behavioral procedures used in the stress-avoidance neurocircuitry literature, there are some recurring findings. The involvement of different subregions of the amygdala is apparent in studies using the predator odor paradigm and ETM. Whereas the former demonstrated increased activity in CeA in avoider animals, the latter showed that acute stress involves an activation of the BLA, while chronic stress enhances activity in the entire amygdala. In contrast, Shukla and Chattarji (2021) showed that chronically stressed animals lack the BLA activation that seems to be required for an appropriate avoidance response in the linear track task. Another brain region that emerges repeatedly is the DRN, the activation of which typically appears to be associated with avoidance (in the ETM or lever press avoidance) (de Andrade et al., 2012, 2013; Hashimoto et al., 2021). Overall, the amygdala (CeA and BLA especially) and DRN appear to be important pivotal regions in the neural circuit that mediates the modulation of avoidance responses after stress induction.

## Discussion

Over the past decades, there has been a rising interest in the effects of stress on avoidance behavior, fueled by clinical and experimental observations suggesting that stress may influence avoidance. On the one hand, there is a clinical intuition that uncontrollable and/or unpredictable stress may ultimately lead to increased avoidance, a hallmark of anxiety-related disorders (Zinbarg et al., 2022). On the other hand, learned helplessness, which is thought to be a model of depression and anxiety-related disorders, is characterized by poor avoidance or escape responses (Maier, 2001). The goal of this review was to synthesize the state of the art regarding the question if and how stress modulates avoidance.

Our main conclusion regarding active avoidance is that prior acute or chronic stress has led to conflicting results. Initially, reports suggested that acute stress impaired active avoidance acquisition (Seligman et al., 1975; Weiss and Glazer, 1975; Lehmann et al., 1999) in the shuttle box, but recent studies challenge this finding, reporting increased active avoidance in the shuttle box (Koba et al., 2001) and also in a lever press avoidance task (Brennan et al., 2005). Regarding chronic stress, we find studies that either report an impairment of acquisition of active avoidance in female rats (Gamaro et al., 1999), no differences between stress or control animals (Weiss et al., 1975) or an increase of active avoidance in male animals (Hata et al., 1989).

Similarly, studies assessing acute effects of stress on passive avoidance report conflicting results. Possible reasons may be that stress duration (Nooshinfar et al., 2011) and individual differences (Navarro-Francés and Arenas, 2014) seem to play a role. A couple of studies compared the effects of acute stress prior to avoidance training, after avoidance training or prior to test to explore if there were differential effects on avoidance acquisition versus expression. One study found that foot shock stress shortly before or after training or prior to test elevated step-through passive avoidance, but had no effect if acute stress was delivered 2 to 3 h before or after training (Jodar et al., 1996). However, another study found that acute stress before or after passive avoidance training resulted in an impairment of avoidance acquisition compared to controls, but these effects disappeared if the stressor was applied 4 h before or 3 h after the avoidance training session (Klenerová et al., 2003a). The majority of the reviewed studies on passive avoidance, especially step-through passive avoidance, support that its acquisition is impaired by chronic stress. However, some studies found no effects (Jodar et al., 1996; Gamaro et al., 1999; Kozlova et al., 2021) or the opposite result (Nooshinfar et al., 2011; Monleón et al., 2015). Studies using the ETM task report that chronic stress increased avoidance in this task (Wood et al., 2008; de Andrade et al., 2013), which contradicts results found using step-through passive avoidance. It may be important to note that avoidance responses in the ETM setting reflect innate behavior, while step-through avoidance requires the acquisition of a counter-instinctive instrumental response (avoidance of the dark), which could potentially explain the conflicting results.

Possible reasons for all these discrepant findings in the literature, apart from differences in stress induction, are manifold. Due to the nature of active and passive avoidance tasks, the former requires that the animal performs a specific response, while in passive avoidance an aversive event occurs unless an animal refrains from performing an action (Krypotos et al., 2015), and this difference between performing a response or refraining from taking action could be one of the reasons. Additionally, most active avoidance tasks require an animal to shuttle between compartments or to press levers when a warning signal is presented, but they do not provide a permanent safe place because the rodent remains in a place where it has received a shock during initial training (Diehl et al., 2019), which is not the case in passive avoidance. This could be another argument for finding conflicting results after stress induction. Nevertheless, these reasons are speculative and should be formally tested. A more recently developed task that does not present the latter limitation (i.e., lack of a safe space in active avoidance procedures) is the platform-mediated avoidance paradigm (Bravo-Rivera et al., 2014). Rodents learn that they can avoid a signaled foot shock if they step onto a platform, which is a safe location. An additional advantage of this task is that avoidance has a cost. If the food-deprived animal decides to step onto the platform it gives up the chance to get a food reward through a lever on the opposite side of the cage. This costly avoidance may better mirror the behavior of an individual with clinical anxiety who may exhibit persistent avoidance despite a loss of potential rewards or positive outcomes (Diehl et al., 2019). To date, there are no published studies assessing the effects of stress in the platform-mediated avoidance paradigm, so the added value of the task in stress research remains to be demonstrated.

In addition to the decades-long research into the behavioral effects of stress on avoidance, more recently, considerable advances have been made in elucidating which neurobiological mechanisms mediate the effects of stress on active or passive avoidance. Putting all studies together, noradrenaline and serotonin neurotransmitter systems appear consistently in the literature, as well as brain structures like the amygdala and the DRN. It will be worthwhile to further unravel how these systems are involved in the mediation of avoidance behavior in stressed subjects. At the same time, it is clear that future studies will need to carefully consider their behavioral assays and take into account that their parametric choices are far from trivial, as highlighted by the current literature review. Researchers will have to carefully consider which duration and type of stressors are optimal to answer their research questions, when to apply the stressor in relation to the avoidance task (e.g., before or after acquisition, or before test), and what animal strain and age to use. They should also consider the inclusion of both sexes, given the opposite findings that have been reported in the literature (Shansky and Murphy, 2021). Tracking behavior and neural activity simultaneously during avoidance acquisition and expression, combined with circuit dissection techniques, could help to unravel the important neurobiological mechanisms that are involved in arbitrating the effects of stress on avoidance (Ball and Gunaydin, 2022).

Another possible avenue where there may be much to gain is the study of individual differences between animals with regard to how their avoidance behavior is shaped by stress. It may be interesting to compare susceptible and resilient animals since not all animals are affected equally by a certain stress induction (e.g., see Duque et al., 2017). Even analyses at the individual level could shine light onto some of the contradictions highlighted in this review. A bottleneck for such individual analysis approaches may be the small sample sizes that are typically used in animal studies, but with the rise in open science and data sharing between labs this should become increasingly feasible. In order to promote collaborations and data pooling, it will be important to include detailed reports of sample sizes, age at arrival [transportation at a young age can be an additional stress factor (Laroche et al., 2009; Holliday et al., 2020)], husbandry conditions, and order and amount of behavioral testing [for a review see Butler-Struben et al. (2022)]. A tool that may help with appropriate reporting are the ARRIVE guidelines (Percie du Sert et al., 2020). Finally, future research could also benefit from a systematic approach regarding stress induction protocols (i.e., agreeing to perform restraint stress for a fixed number of days during a certain amount of hours, to be able to more readily compare studies) and to build up from previous work. As discussed above, the large procedural variability in the current literature could easily drive different results, making it hard to interpret it as a whole.

In conclusion, although there is abundant evidence that stress can affect avoidance, it is clear that there is still much to know on how exactly acute and chronic stress influences passive and active avoidance, as well as which molecular pathways and brain areas are involved in mediating these effects. Further progress on these matters will be indispensable for a better understanding of the role of stress in the development of the persistent avoidance that characterizes clinical anxiety and other mental health conditions.

## Author contributions

AL-M performed the literature search and wrote the first draft. TB and LL edited and critically reviewed the manuscript. All authors have made a substantial, direct, and intellectual contribution to the conception and writing of this work, and approved it for publication.
